# Post-traumatic rhabdomyolysis: an observational study in seven patients

**DOI:** 10.1186/cc11065

**Published:** 2012-03-20

**Authors:** M Alezrah, A Berger, P Bentzinger, C Sassot, L Profumo, B Saumande, O Collange, A Meyer, B Calon

**Affiliations:** 1 réanimation chirurgicale, Strasbourg, France

## Introduction

In the ICU, post-traumatic rhabdomyolysis is a relatively rare (1/5) cause of crush syndrome [1]. Early aggressive treatment is quintessential to avoid complications such as renal failure and death [2]. This observational study intends to assess the incidence of complications after traumatic crush injury in a tertiary trauma center ICU.

## Methods

During 24 months, seven patients admitted to our surgical intensive care after polytrauma (ISS >15) suffered severe rhabdomyolysis (CPK >5,000 U/l) treated by intensive fluid resuscitation, bicarbonate and furosemide.

## Results

The following data are reported in Table 1: renal function (initial creatinine, renal replacement therapy (RRT), rhabdomyolysis (maximal CPK and myoglobin), acidosis (lowest pH, highest lactate (HL), time lactate >5 mmol/l) and complications (mortality, neurological sequelae).

**Table 1:** Results

Initial creatinine (μmol/l)	69 to 198
RRT	2/7
Maximal CPK (10^3 ^U/l)	11 to 144
Maximal myoglobin (10^3 ^U/l)	4 to 159
pH	7 to 7.3
Highest lactate (mmol/l)	2 to 28
Time lactate >5 mmol/l (hours)	0 to 84
Mortality	0
Neurological sequelae	6/7

## Conclusion

Survival was 100% but neurological impairment in the limbs is a major complication. The two RRT patients had a wide range of maximal CPK levels (15,780 to 52,600 U/l), but more severe acidosis (lowest pH 7.0 to 7.2, maximum lactate: 7.5 to 28 mmol/l, acidosis duration: 72 to 84 hours). This acidosis turned out to be due to intra-abdominal complications: post-traumatic pancreatitis and mesenteric ischemia. The vital prognosis of post-traumatic crush injury was good but the sequelae of the compartment syndrome were major. The need for RRT was not linked to CPK levels but rather to acidosis due to intra-abdominal complications.
